# In Vivo Tagging and Characterization of S-Glutathionylated Proteins by a Chemoenzymatic Method**

**DOI:** 10.1002/anie.201200321

**Published:** 2012-05-03

**Authors:** Bing-Yu Chiang, Chi-Chi Chou, Fu-Tan Hsieh, Shijay Gao, Jason Ching-Yao Lin, Sheng-Huang Lin, Tze-Chieh Chen, Kay-Hooi Khoo, Chun-Hung Lin

**Affiliations:** Institute of Biological ChemistryAcademia Sinica No. 128, Academia Road Section 2, Nan-Kang, Taipei, 11529 (Taiwan) and Institute of Biochemical Sciences National Taiwan University (Taiwan)

**Keywords:** chemoenzymatic labeling, glutathionylation, protein modifications, proteomics, redox chemistry

Glutathione (GSH), a sulfhydryl-containing tripeptide present in most organisms at millimolar levels, plays a crucial role in redox homeostasis.^1^ Reactive cysteine residues are vulnerable to reactive oxygen or nitrogen species and thus depend heavily on GSH to avoid irreversible oxidation.^[1a]^, ^[2]^ Reversible conjugation of GSH to proteins through the formation of mixed disulfide bonds is termed protein glutathionylation (PSSG), which additionally alters or regulates protein functions in biological processes, including energy metabolism, signal transduction, ion transport, cytoskeletal assembly, and protein folding.^[2a]^ Although various possible mechanisms have been proposed for PSSG,^[1a]^ the delineation of its functional consequences in vivo remains a longstanding challenge owing to lack of appropriate tools to globally identify this important modification with high sensitivity.^[3]^

A conventional method to detect PSSG relies on the metabolic labeling of endogenous GSH with radioactive ^35^S-cysteine and subsequent phosphor imaging on the gel obtained from 2D PAGE.^[4]^ However, this approach does not distinguish different types of S-thiolation, for example, cysteinylation from glutathionylation. Another challenge is the shortage of straightforward methods to enrich and identify these modified proteins.^[5]^ Although commercially available anti-GSH antibodies have been applied to detect PSSG, serious concern has been raised regarding their specificity and sensitivity.^[5]^ Recently, glutaredoxin-dependent biotin-switch methods were developed for efficient detection and enrichment of GSH-modified proteins.^[6]^ This method, however, requires alkylation of reduced cysteine residues, followed by reduction of GSH-modified cysteines with bacterial glutaredoxin and subsequent biotin labeling. Several concerns have been raised, such as incomplete reduction and alkylation, the specificity of bacterial glutaredoxin, and possible oxidations in cysteine residues of proteins owing to ambient exposure. Biotinylated GSH disulfide (biotin-GSSG) has also been used to directly label protein cysteine residues.^[7]^ The membrane permeability of biotin-GSSG enables intracellular formation of mixed sulfides. Although this method is suitable for detection and enrichment, the external addition of biotin-GSSG likely alters the normal thiol content and the GSH/GSSG ratio given that GSSG usually accounts for less than 1 % of the total GSH content in mammalian cells.^[1a]^ Furthermore, since the action of GSSG is not the major route of protein glutathionylation under physiological conditions, such a method may exclude an unexpectedly large pool of proteins during analysis.

Herein we developed a selective and rapid method for detecting and identifying PSSG in mammalian cells by using biotinyl spermine (biotin-spm) and *E. coli* glutathionylspermidine synthetase (GspS; Figure 1 a, b).^[8]^

**Figure 1:**
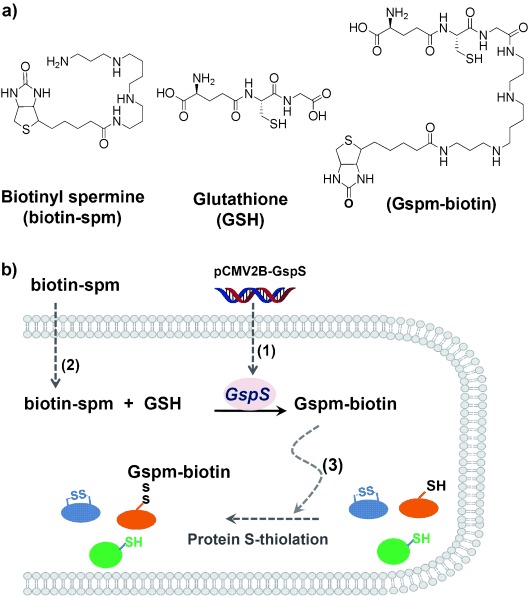
a) The structures of biotin-spm, GSH, and Gspm-biotin. b) A schematic diagram depicting intracellular labeling of glutathionylated proteins by incorporation of Gsp synthetase (GspS) and biotin-spm. (1) The gene of GspS is transfected into 293T cells. (2) After the biotin-spm uptake, expressed GspS is then able to convert intracellular GSH to Gspm-biotin. (3) Gspm-biotin conjugates to reactive cysteine residues of proteins.

The enzyme GspS catalyzes the amide bond formation between GSH and spermidine to generate glutathionylspermidine (Gsp; Figure 1 in the Supporting Information), a unique GSH derivative found only in a few Gram-negative bacteria and protozoa.^[8]^ We previously discovered that Gsp behaves similarly to GSH in forming disulfide bonds with cysteine residues of proteins in vivo.^[9]^ Consequently, if the enzyme GspS is expressed in mammalian cells and generates Gsp, the mixed-disulfide bond formation between Gsp and protein cysteine residues would be akin to in situ protein glutathionylation.

The DNA fragment of the GspS, corresponding to the amino acid sequence of the Gsp synthetase domain, was incorporated to the pCMV2B vector to construct pCMV2B-GspS. The introduction of the vector pCMV2B-GspS into human embryonic kidney (HEK) 293T cells allowed expression of the enzyme GspS and the subsequent conversion of endogenous GSH to Gsp in vivo (Figure 2). Based on the structure of the enzyme GspS in complex with substrate (PDB code: 2IOA),^[10]^ one NH_2_ group of spermidine is near the surface and freely available for additional chemical derivatization. Computational modeling suggested that the biotin moiety of biotin-spm likely protrudes into the solvent and does not participate in substrate binding (Figure 2 in the Supporting Information). We therefore designed and synthesized biotin-spm as the probe to tag GSH (Scheme 1 in the Supporting Information). The Michaelis–Menten kinetics parameters indicate that biotin-spm (Michaelis–Menten constant *K*_m_ and turnover number *k*_cat_ of 74 μm and 2.7 s^−1^, respectively, Figure 3 in the Supporting Information) is comparable to the native substrate spermidine (*K*_m_ and *k*_cat_ of 76 μm and 4.6 s^−1^, respectively).^[10]^ The morphology of viable 293T cells and the MTT (3-(4,5-dimethylthiazol-2-yl)-2,5-diphenyl tetrazolium bromide) assay indicated that biotin-spm does not dramatically alter cell viability. Neither biotin-spm nor biotinylated glutathionylspermine (Gspm-biotin, the enzyme reaction product) caused considerable cytotoxicity (Figure 4 in the Supporting Information).

**Figure 2:**
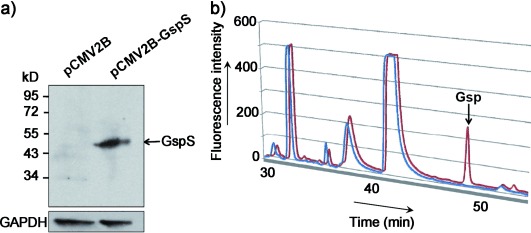
a) Vectors pCMV2B (control) and pCMV2B-GspS were separately delivered into 293T cells. The expression of the enzyme GspS in 293T cells was detected by an anti-FLAG antibody. The arrow corresponds to GspS (MW=51 kDa). D-Glyceraldehyde-3-phosphate dehydrogenase (GAPDH) was used as a loading control. b) The enzyme GspS converts intracellular GSH to Gsp in 293T cells. Cell lysates were treated with monobromobimane (mBBr), and the Gsp level was analyzed by HPLC, where mBBr-derivatized thiol compounds were detected by fluorescence (excitation/emission: 394 nm/390 nm). The arrow corresponds to the signal of mBBr-derivatized Gsp. HPLC traces for cells transfected with vectors pCMV2B (blue) and pCMV2B-GspS (red) are shown.

We next incubated *gsps*-transfected 293T cells with biotin-spm for 36 h and then detected PSSG in cell lysates by nonreducing immunoblotting with an anti-biotin antibody. The presence of intracellular protein biotinylation indicated that biotin-spm is cell permeable, and that Gspm-biotin molecules have formed mixed disulfides with protein cysteine residues (Figure 3 a). The biotin signal was almost compeletly abolished when the cell lysates were treated with 2-mercaptoethanol or Gsp amidase, which catalyzes cleavage of the amide bond between GSH and spermidine. This observation supported the idea that disulfide bonds are formed between Gspm-biotin and cysteine residues of proteins (Figure 3 b). The addition of H_2_O_2_ or diamide to cell cultures significantly enhanced the levels of biotinylation (Figure 3 c), a finding that is consistent with the observation that oxidative stress increases protein glutathionylation.^[4]^, ^[11]^

**Figure 3:**
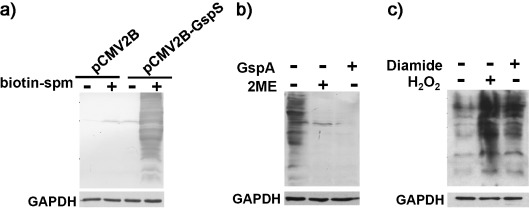
Detection of protein Gspm-biotin S-thiolation by immunoblotting. Anti-biotin antibody is used to indicate the presence of Gspm-biotin S-thiolation on proteins; GADPH serves as a gel loading control. a) Only *gsps*-transfected cells treated with biotin-spm are positive for biotinylation. b) Levels of Gspm-biotin S-thiolation in *gsps*-transfected cells treated with biotin-spm are dramatically reduced upon reduction by 2-mercaptoethanol (2ME) or removal of biotin-spm by Gsp amidase (GspA), thus indicating that S-thiolation by Gspm-biotin is the major mode of protein biotinylation. c) After addition of H_2_O_2_ or diamide to *gsps*-transfected cells treated with biotin-spm, levels of Gspm-biotin S-thiolation increase significantly.

To identify glutathionylated proteins and their modified cysteines, the *gsps*-transfected 293T cells were incubated with biotin-spm (2 mm) for 24 h and then with H_2_O_2_ (1 mm) for five minutes. Iodoacetamide (IAM, 50 mm) was used to cap free thiols that may otherwise be mistakenly identified in later analysis. Acetone was added to precipitate proteins and to remove unreacted biotin-spm and Gspm-biotin. The precipitate was dissolved with trifluoroethanol and digested with trypsin for 20 h. Gspm-biotin S-thiolated peptides were captured by streptavidin-conjugated resin. After several washes and elution, these target peptides were treated with Gsp amidase (for the removal of biotin-spm) to generate glutathionylated peptides for LTQ-Obitrap LC–MS/MS analysis (Figure 4).

**Figure 4:**
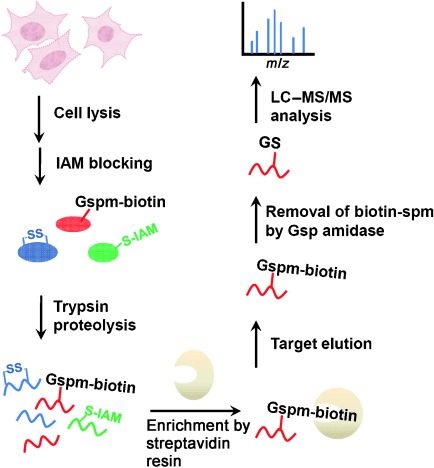
The workflow for site-specific identification of protein glutathionylation in *gsps*-transfected 293T cells. Cells are lysed and treated with iodoacetamide to block free thiols. After tryptic digestion, Gspm-biotin S-thiolated peptides are enriched by streptavidin resin, and subsequently hydrolysis by Gsp amidase leads to removal of biotin-spm, thereby leaving intact glutathion on labeled peptides. Samples are then analyzed by LC–MS/MS.

We identified 1409 unique glutathionylated cysteine residues from 913 proteins in 293T cells in each of two independent experiments (Data 1 in the Supporting Information). Among them, 49 proteins were identified in previous proteomics studies (highlighted in Data 1 in the Supporting Information), thus suggesting that those proteins may be sensitive to the redox environment in various cell types. However, for these 49 matches no site information was obtained in earlier studies, owing to the methods used (e.g. 2D-PAGE and gel-assisted mass spectrometric analysis). Some of the proteins, including glutathione S-transferase pi (GSTπ) and sarco/endoplasmic reticulum Ca^2+^-ATPase (SERCA),^[12]^ are known to be modified by GSH. The glutathionylated cysteines of these proteins are consistent with those of previous studies (Table 1). For example, GSTπ has four cysteine residues, the Cys48 and Cys102 of which were reported to be glutathionylated under oxidative stress.^[12a]^ Both cysteine residues were unambiguously shown to be glutathionylated in our studies given the presence of their distinctive fragment ions in the corresponding tandem mass spectra. (Figure 5 a, b) These observations suggested that Gspm-biotin can function as does GSH and can form mixed disulfides with cysteine residues of proteins in vivo. Few reported glutathionylated proteins, including protein tyrosine phosphatase 1B^[13]^ and endothelial nitric oxide synthase,^[14]^ however, were not found in our study, probably owing to the relatively low abundances and localization on the membrane.

**Figure 5:**
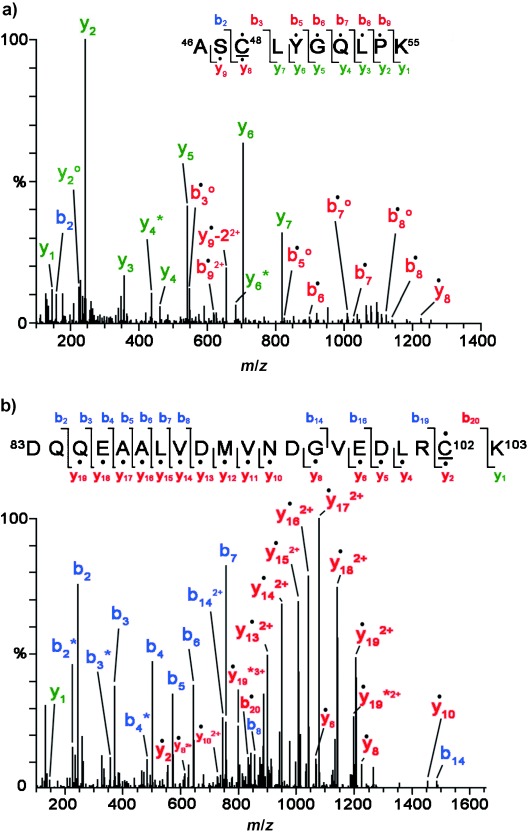
MS/MS spectra derived from two glutathionylated tryptic peptides of glutathione S-transferase pi (GSTπ), which afford the [*M*+2 H]^2+^ precursor ion at *m*/*z* 692.82 for the Cys48-containing peptide (a) and the [*M*+3 H]^3+^ precursor ion at *m*/*z* 885.05 for the Cys102-carrying peptide (b). The amino acid sequences and respective b and y ions are shown in each spectrum, with the glutathionylated cysteine residues underlined. Filled circles (dots) above the labeled ions indicate product ions carrying a GSH moiety, which contributes to a mass increment of 305 Da relative to those ions without modification. Open circles (○) and asterisks (✶) indicate product ions after a neutral loss of H_2_O and NH_3_, respectively. “−2” on y ion y_9_ indicates the presence of a cysteine thioaldehyde residue, the molecular weight of which is 2 Da lower than that of cysteine.

**Table 1:** A subset of glutathionylated proteins identified herein and their corresponding sites of modification that were reported. The glutathionylated cysteines are shown in red.

Names of the proteins ^[Ref.]^	Identified peptide sequences
Glutathione S-transferase pi^12a^	DQQEAALVDMVNDGVEDLRCK, ASCLYGQLPK
Actin, cytoplasmic 2^17^	LCYVALDFEQEMATAASSSSLEK
Heat shock protein 60 kDa^17^	CIPALDSLTPANEDQK
Elongation factor 1-alpha 1^17^	SGDAAIVDMVPGKPMCVESFSDYPPLGR
Thioredoxin^18^	CMPTFQFFK
Creatine kinase^19^	LGYILTCPSNLGTGLR
Cellular tumor antigen p53^20^	SVTCTYSPALNK
Isocitrate dehydrogenase^21^	SEGGFIWACK
Peroxiredoxin 1^22^	HGEVCPAGWKPGSDTIKPDVQK
Superoxide dismutase [Cu–Zn]^23^	HVGDLGNVTADKDGVADVSIEDSVISLSGDHCIIGR
SERCA^12b^	SMSVYCTPNKPSR
Catechol O-methyltransferase^24^	GTVLLADNVICPGAPDFLAHVR YLPDTLLLEECGLLR
Annexin A2^25^	GLGTDEDSLIEIICSR

In addition to the proteins involved in redox regulation, we identified numerous glutathionylated proteins that are involved in various cellular activities, for example signal transduction (cAMP-dependent protein kinase), cell cycle (E2 ubiquitin ligase/E3 ubiquitin hydrolase), energy metabolism (glyceraldehyde-3-phosphate dehydrogenase), and cytoskeleton assembly (actin). These results support the idea that PSSG likely affects various protein functions.^[2a]^ Glutathionylation masks reactive cysteines, which may abolish protein function.^[2a]^ In certain cases, however, glutathionylation enhances (e.g. SERCA) or does not alter the function of a protein.^[2a]^, ^[12b]^ The ability to pinpoint the specific residues modified by glutathionylation may offer useful insight into the structural and functional alterations that are associated with cellular regulation.

Although glutathionylated proteins have been identified in various cell types, none of the available methods are able to detect the attached GSH to provide definitive evidence of site-specific glutathionylation.^[5]^ Furthermore, the fragmentation efficiency of a biotin tag is often too low to obtain site-specific information in an MS/MS spectrum.^[15]^ Our probe not only facilitates the purification of S-thiolated proteins, but also can be enzymatically removed before MS analysis. Particularly, the enzymatic labeling of GSH by the enzyme GspS can distinguish the intramolecular (protein disulfide bonds) and different types of intermolecular disulfide linkages (glutathionylation and cysteinylation). Our method preserves the GSH modification during enrichment and thus largely eliminates the false positive results that are commonly introduced by the other methods and avoids the need for sequencing algorithms to account for neutral losses caused by collision-induced fragmentation.^[16]^ Those features contributed favorably to our ability to identify more PSSG at a large scale, as well as to successfully determine the GSH S-thiolation sites in most cases.

Our strategy reported herein is the first to label the endogenous GSH with an engineered tag to allow sensitive detection as well as selective purification of glutathionylated proteins. Since spm-biotin can be independently added to the cell culture expressing the enzyme GspS, our approach requires no external dosing of GSH derivatives that may alter the cellular thiol content and the GSH/GSSG ratio. Moreover, the use of Gspm-biotin also requires no reduction of oxidized cysteines, such as sulfenic acid and nitrosocysteine, otherwise necessary for biotin switches. Therefore, our approach is complementary to the recent approaches to dissect the process of PSSG under physiological condition, as well as to decode the constitutive S-glutathionylation (Figure 3 c).

In summary, we developed an efficient chemo-enzymatic approach for probing glutathionylated proteins by introducing *E. coli* GspS into mammalian cells for labeling intracellular GSH. By combining enrichment and MS analysis, our method enables to site-specifically pinpoint S-thiolated residues. The quantitative measurement, currently in progress, will further expand the utility of our methods to better define the functional consequences of PSSG.

## Supporting Information

Detailed facts of importance to specialist readers are published as ”Supporting Information”. Such documents are peer-reviewed, but not copy-edited or typeset. They are made available as submitted by the authors.
