# Supplementary material for: In Vivo Tagging and Characterization of S-Glutathionylated Proteins by a Chemoenzymatic Method
Source: Angew Chem Int Ed Engl. 2012 May 3;51(24):5871–5. doi: 10.1002/anie.201200321 (PMC3505901; doi:10.1002/anie.201200321)
Supplement: Supplementary file 1 [file anie0051-5871-sd1.pdf]

Angewandte Chemie
Eine Zeitschrift der Gesellschaft Deutscher Chemiker

Supporting Information
© Wiley-VCH 2012
69451 Weinheim, Germany

In Vivo Tagging and Characterization of S-Glutathionylated Proteins by a Chemoenzymatic Method**

Bing-Yu Chiang, Chi-Chi Chou, Fu-Tan Hsieh, Shijay Gao, Jason Ching-Yao Lin, Sheng-Huang Lin, Tze-Chieh Chen, Kay-Hooi Khoo, and Chun-Hung Lin**

anie_201200321_sm_miscellaneous_information.pdf

Supporting Information Figure 1. The reactions catalyzed by *E. coli* glutathionylspermidine synthetase/amidase. Glutathionylspermidine synthetase/amidase is a bifunctional enzyme with an amidase domain at the N terminus and a C-terminal synthetase domain.

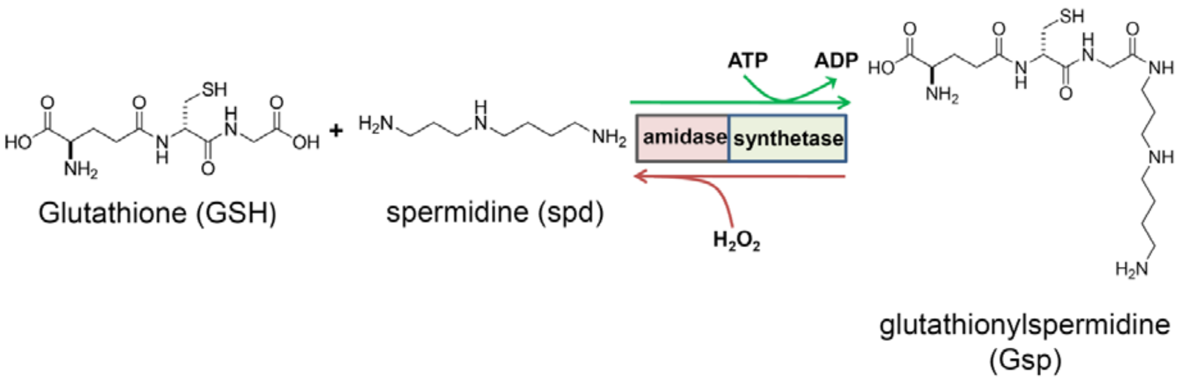

Supporting Information Figure 2. The biotin moiety of biotin-spm protrude into the solvent and does not participate in substrate binding Biotin-spm and GSH are docked into the active site of GspS (PDB ID:2IOA). The model was created using PyMOL. The carbon, oxygen and nitrogens atoms of biotin-spm and GSH are represented in green, red and blue, respectively. Protein carbon, oxygen, and nitrogen atoms, are shown in grey, red and blue, respectively.

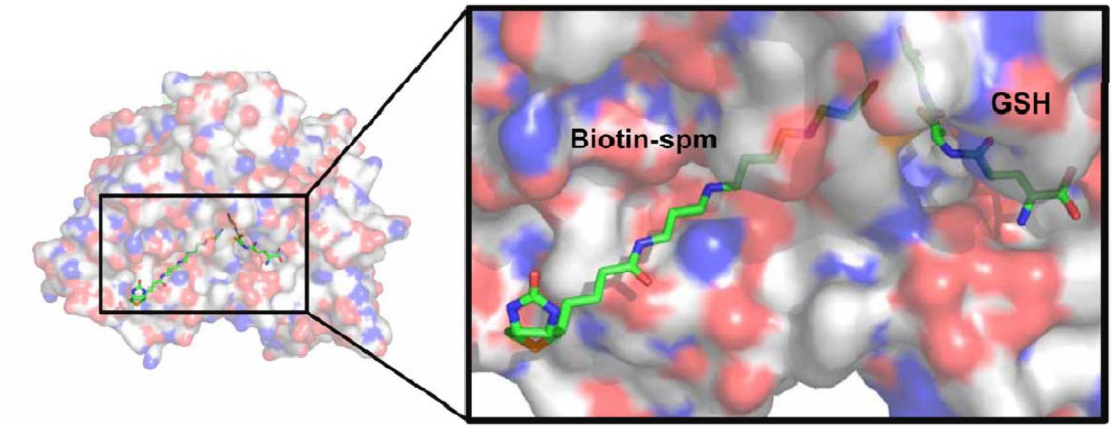

Supporting Information Figure 3. A plot of V_0 versus biotin-spm concentration to determine K_m and k_{cat} values Reaction rate (V_0) was plotted against the concentration of biotin-spm. To confirm whether biotin-spm is a substrate for Gsp synthetase, recombinant Gsp synthetase were incubated with various concentrations of biotin-spm and the resulting activities were assessed by measuring the consumption of NADH by the pyruvate kinase/lactate dehydrogenase-coupled assay. The K_m and k_{cat} values of biotin-spm were determined by a previously reported spectrometric assay and found to be similar to those of the native substrate spermidine (K_m = 76 µM and k_{cat} = 4.6 s^{-1}).

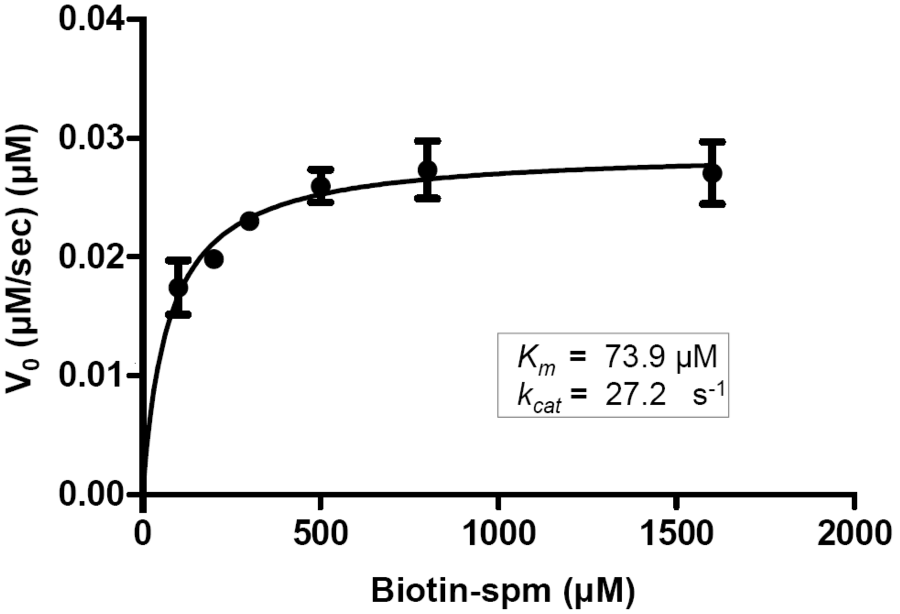

Supporting Information Figure 4. Biotin-spm does not cause cytotoxicity to 293T cell. a) The morphology of viable 293T cells, examined by light microscopy at 200× magnification on a Nikon Eclipse TE2000-U stereo microscope with a digital camera attachment. The cells appeared to be unaffected by treatment with 1 mM biotin-spm for 24 h. **b)** The MTT (3-(4,5-dimethylthiazol-2-yl)-2,5-diphenyl tetrazolium bromide) assay indicated that biotin-spm (up to 10 mM) does not alter cell viability. The cells without biotin-spm treatment was normalized as 100% .

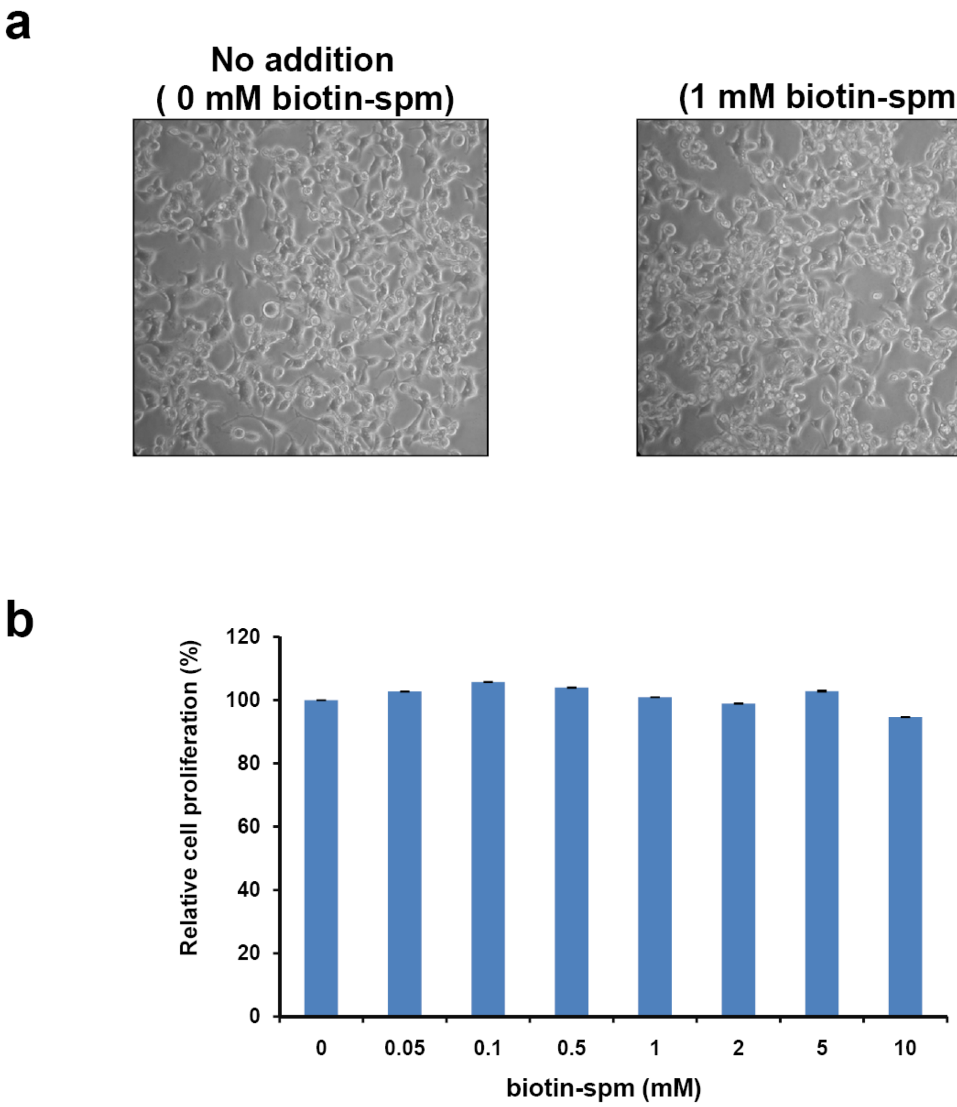

Supporting Scheme 1. Synthetic procedure of biotinyl-spermine

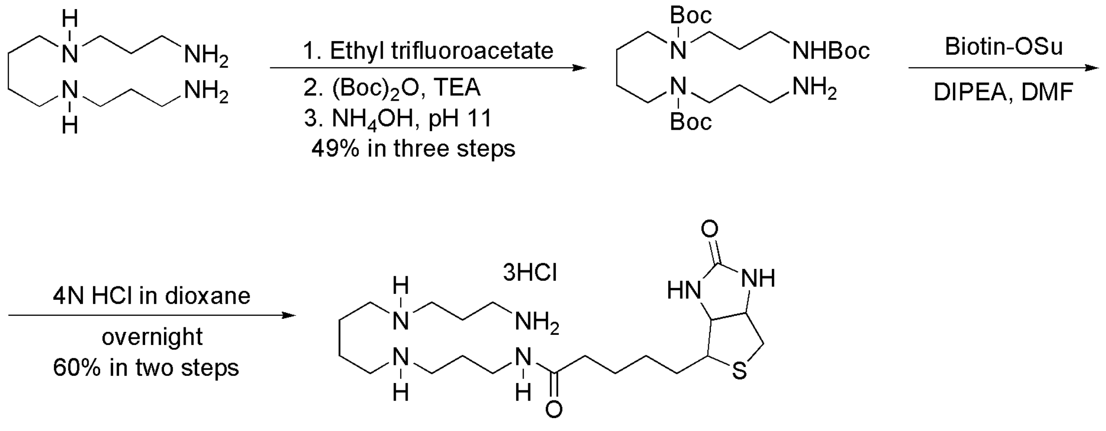

Experimental Section

Reagents and Chemicals

Rabbit anti-GAPDH and goat HRP-conjugated anti-mouse antibodies were purchased from Abcam and Chemicon, respectively. Mouse alkaline phosphate (AP)-conjugated anti-biotin and mouse anti-FLAG antibodies were purchased from Sigma. Immunostaining reagents such as CDP-Star Chemiluminescence reagents for AP or Western ECL reagents for HRP were purchased from Perkin-Elmer. Other chemicals were purchased from Sigma and Merck unless specified otherwise.

Synthesis of Biotinyl-spermine (biotin-spm)

The synthetic scheme is provided in supporting scheme 1. Spermine (2.0 mmol) was dissolved in excess MeOH under N_2 and the solution was then cooled to -78 ^{o}C, after which ethyl trifluoroacetate (2.0 mmol) was added dropwise over 0.5 h. The solution stirred for an additional 0.5 h before the temperature was raised to 0 ^{o}C over the next 1 h. Di-*tert*-butyl dicarbonate (8.0 mmol) in MeOH (20 ml) was added to the solution over 10 min at 0 ^{o}C, and stirred overnight at room temperature to protect the amino groups of spermine. The resulting mixture, without separation, was pH-adjusted to above 11 by the addition of concentrated $NH_4OH_{(aq)}$ and then stirred overnight at room temperature. The resulting mixture was evaporated and purified by silica gel chromatography with $MeOH/CHCl_3/NH_4OH$ (from 10/70/1 to 10/50/1), leading to a pale-yellow oily product (49% yield). The NMR ^{1}H and HRMS spectra were consistent with those previously reported[12].

Biotin *N*-hydroxysuccinimide ester (1.58 mmol) in dry DMF is added to a suspension of N^1,N^2,N^3-tri-Boc-spermine (1.32 mmol) in dry DMF, followed by the addition of diisopropylethylamine (1.58 mmol). The mixture was stirred overnight at room temperature and evaporated under high vacuum, and redissolved in ethyl acetate; the organic phase was sequentially washed with saturated $NaHCO_3$(aq), 0.5N HCl(aq), dried over $MgSO_4$, and concentrated under reduced pressure to give a glassy solid. Without purification, the aforementioned solid was redissolved in dry dioxane and treated with 4 N HCl in dioxane (20 ml) at 0 ^{o}C, followed by stirring overnight at room temperature. The resulting mixture was filtered and the filtrate was recrystallizated from $MeOH/Et_2O$ to give a white powder (0.42 g, 60%). Product characterizations are as follows: $[\alpha]^{20}_{D}$ +32.5 (*c* 1.66, H_2O); TLC ($MeOH/CHCl_3/NH_4OH$ = 2/1/1), R_f 0.4; ^{1}H NMR (500 MHz, D_2O) 1.38–1.56 (m, 2H), 1.57-1.82 (m, 4H), 1.82-1.90 (m, 4H), 1.96 (tt, J = 7.8, 7.2 Hz, 2H), 2.12-2.19

(m, 2H), 2.34 (t, J = 7.3 Hz, 2H), 2.85 (d, J = 13.1 Hz), 3.06 (dd, J = 13.0, 5.0 Hz, 1H), 3.10-3.24 (m, 10H), 3.36 (t, J = 6.7 Hz, 2H), 3.41 (m, 1H), 4.49 (dd, J = 7.9, 4.5 Hz, 1H), 4.7 (dd, J = 7.9, 4.7 Hz, 1H); ^{13}C NMR (125 MHz, D_2O) 177.3, 165.3, 62.0, 60.2, 55.3, 46.9, 46.8, 45.0, 44.4, 39.6, 36.5, 35.8, 35.3, 27.9, 27.6, 25.6, 25.0, 23.6, 22.7; HRMS (ESI) calcd for $C_{20}H_{40}N_6O_2S$ $[M+H]^+$ 429.3006, found 429.2996

Construction and transfection of pCMV2B-GspS

The primers used for PCR amplification, 5'-CAGCTGCAGATTGCAGGCGAGCTGCTGAAAATC and 5'- GCTCAAGCTTTTACTTTTTCACCACAATTAACGGTTCAATATCAC were designed to generate the DNA sequence of Gsp synthetase (corresponding to the amino acid sequence 204~619 of the bifunctional protein, Gsp synthetase/amidase). The product DNA (corresponding to Gsp synthetase domain) was inserted into the vector pCMV2B (Stratagene) via restriction sites *PstI* and *HindIII*. The plasmid pCMV2B-GspS was delivered via Lipofectamine 2000 (Invitrogen) following the manufacturer's recommended protocols. Briefly, 10^6 cells were cultured into a 60 mm tissue culture dish (Corning) prior to transfection. The growth medium was replaced by serum-free DMEM for 4 h. 8 μg of pCMV2B-GspS was added to serum-free DMEM to make up a final volume of 500 μl, and 20 μl of Lipofectamine 2000 was pipetted into a separate tube of serum-free DMEM for a final volume of 500 μl. Both the plasmid and Lipofectamine were carefully and individually mixed, allowed to set for 5 min, and then mixed together. The mixtures was incubated for 20 min at room temperature to form plasmid containing liposome and then 1 ml of the mixture was slowly added dropwise into the cell culture to prevent cell detachment. The culture was carefully agitated to ensure proper dispersion and then incubated for 4 h at 37 °C with 5% CO_2 before the medium was replaced by serum-containing DMEM.

HEK 293T cell culture

HEK 293T cells were cultured in DMEM (Invitrogen) supplemented with 10% fetal bovine serum (FBS, Biochrom), 1% penicillin/streptomycin/glutamine and 1% sodium pyruvate (Gibco, Invitrogen) at 37 °C, 5% CO_2 throughout the experiment.

***In vivo* labeling and immunoblotting of Gsp-biotin in 293T cell**

293T cells were subcultured into 10 cm tissue culture dishes (Corning) and transfected with the pCMV2B-GspS plasmid as previously described. After 4 h of liposome treatment, the culture media were replenished with FBS-supplemented DMEM spiked with 1 mM biotin-spm for 24 h at 37 °C, 5% CO_2 and treated with 1 mM diamide or H_2O_2 for 10 min to enhance the level of protein glutathionylation,

followed by detachment by PBS and centrifugation at 500 × g (Kobuta) for 5 min. Cells were then sonicated in PBS. After centrifugation to remove cell debris, the resulting samples were resolved in a non-reducing SDS-PAGE. Gluthionylated proteins were detected by Western blotting using mouse alkaline-phosphatase-conjugated anti-biotin antibody. To remove biotin-spm from Gsp-biotin thiolated proteins, 10^7 cells were harvested and lysed by sonication, One half of the resulting lysates were treated with 1 μg Gsp amidase at 37 °C for 1 h, while the other half of cell lysate was treated with the same volume of SDS loading buffer containing 50 mM 2-mercaptoethanol (2-ME) and boiled for 30 min. Both the reaction mixtures were subjected to 10% non-reducing SDS-PAGE and Western blotting using an anti-biotin antibody.

Preparation and purification of GSH-modified peptides

After 4 h of liposomal transfection of pCMV2B-GspS as previously described, the cultures were replenished with FBS-supplemented DMEM spiked with 2 mM biotin-spm for 24 h at 37 °C, 5% CO_2 and treated with 1 mM H_2O_2 for 10 min to enhance the level of gluthionylation, prior to detachment by PBS and centrifugation at 500 × g for 5 min. Approximately 6 × 10^7 cells were collected and resuspended in lysis buffer (50 mM 2-iodoacetamide (IAM), 5 mM EDTA in PBS at pH 7.4). After lysis by sonication, cell lysates were mixed with cold acetone (1:4 v/v) and incubated at –20 °C for 1 h to precipitate proteins. After the precipitates were washed three times with ethanol, trifluoroethanol (TFE) was added to a final volume of 50% to resolubilize and denature proteins in a 60 °C water-bath for 30 min. The sample was diluted with PBS to reduce the amount of TFE to 5% and tryptic-digested at 37 °C for overnight with 20 μg trypsin (of sequencing grade, Promega). Gsp-biotin S-thiolated peptides were enriched by 200 μl of high capacity streptavidin-agarose resin (Thermo) by incubating the mixture for 4 h at 25 °C. Unbound peptides were removed by several washes with 10 ml PBS (pH 7.4), milli Q water and 10% acetonitrile (at least 4 times). Captured peptides were then eluted with 0.4% trifluoroacetic acid and 30% acetonitrile and resolubilized in PBS, followed by treatment with 100 ng Gsp amidase at 37 °C for 30 min to remove Spm-biotin. The resulting peptides were filtered by Nanosep 3 k Omega (Pall) for the removal of Gsp amidase. After drying, samples were analyzed by mass spectrometry.

Mass spectrometry and data processing

The peptide mixtures from each preparation were desalted with C_{18} Ziptips (Millipore), and dried in a SpeedVac (Thermo), before they were reconstituted in 5% acetonitrile and 0.1% formic acid to give a volume of 5 μl, and loaded onto a C_{18}

column of 75-μm × 250-mm (nanoACQUITY UPLC BEH130, Waters). The peptides mixtures were separated by online nanoflow liquid chromatography using nanoAcquity system (Waters) with a linear gradient of 5 to 50% acetonitrile (in 0.1% formic acid) in 95 min, followed by a sharp increase to 85% acetonitrile in 1 min and held for another 15 min at a constant flow rate of 300 nl min^{-1}. Peptides were detected in an LTQ-Orbitrap Velos hybrid mass spectrometer (Thermo Scientific) using a data-dependent HCD Top10 method. For each cycle, full-scan MS spectra (m/z 300–2000) were acquired in the Orbitrap at 30,000 resolution (at m/z 400) after accumulation to a target intensity value of 10^6 ions in the C-trap. The 10 most intense ions with charge states ≥2 were sequentially isolated to a target value of 50,000 ions within a maximum injection time of 250 ms and fragmented by higher-energy C-trap dissociation (HCD) in the octopole collision cell with a normalized collision energy of 35%. The fragment ions were then detected in the Orbitrap at 7,500 resolution. Ion selection threshold was 1000 counts for MS/MS, and the selected ions were excluded from further analysis for 180 s. All MS and MS/MS raw data were processed with Proteome Discoverer version 1.2 (Thermo Scientific), and the peptides were identified from the MS/MS data searched against the UniProtKB/Swiss-Prot (20110531/529056 sequences entries) database using the Mascot search engine 2.3.02 (Matrix Science). Search criteria used were as follows: trypsin digestion; human taxonomy; considered variable modifications of cysteine glutathionylation (+ 305.068156 Da), methionine oxidation (+ 15.9949 Da), and cysteine carboxyamidomethylation (+ 57.0214 Da); up to two missed cleavages were allowed; and mass accuracy of 10 ppm for the parent ion and 0.10 Da for the fragment ions. False discovery rates were controlled using the target-decoy strategy to distinguish correct and incorrect identifications. For the identification, the false discovery rate was set to 0.01 for peptides, proteins and sites. All mass spectrometric data of all glutathionylated proteins and peptides are available online (Supporting Information Data 1).

HPLC analysis of the intracellular Gsp

Transfected (pCMV2B-GspS) and control samples (pCMV2B vector only) were collected from culture dishes in PBS (Gibco) and centrifuged at 1,000 rpm for 5 min. After the supernatant was decanted, the pellet was suspended in a solution of 40mM HEPES (pH 8.0), 2 mM EDTA, 10 mM 2-ME (Baker) and boiled for 20 min. 10 mM monobromobimane (mBBr, Invitrogen) in absolute ethanol was added to the samples in the dark to initiate the labeling reaction. The mixtures were then incubated in a 70 °C water-bath for 3 min to facilitate the reaction and quenched by slow addition of 1.6 M methansulfonic acid (pH adjusted to 1.5 with lithium hydroxide) at 0 °C over the period of 15 min. The resulting mixtures were centrifuged at 15000 × g for 1 h and the

supernatants were filtered through a 0.45 μm Minisart hydrophilic membrane syringe filter (Sartorius) for the subsequent HPLC analysis. Reverse phase C_{18} columns (HiChrom) were used in a gradient of 0.25% w/v lithium camphor sulfate, pH 2.64 (Buffer A), and a solution of 0.25% w/v lithium camphor sulfate, pH 2.64, in 25% v/v *n*-propanol (Buffer B) in a Perkin-Elmer Series 200 HPLC system. During the analysis, the gradient started with 90% Buffer A for 20 min and then changed to, 90%-50% Buffer A for 40 min and 50% Bufer A for 10 min. Equilibration among each sample injection was established by applying 90% Buffer A for 30 min. mBBr fluorescence was measured by excitation at 360 nm excitation and emission at 480nm.

Cell Proliferation Assay

CellTiter 96 AQ One Solution Cell Proliferation Assay (Promega) was used to determine the cytotoxicity of biotin-spm in 293T cells. pCMV2B and pCMV2B-GspS transfected cells were subcultured into 96-welled tissue-culture microtiter plates (Corning) and treated with biotin-spm at concentrations from 0 to 10 mM for 36 h, followed by addition of MTT-containing reagent at a ratio of 1:5 v/v for 1 h at 37 °C, 5% CO_2, before measurement of the formazan absorbance. Cell images were digitally captured with 200-fold magnification by a Nikon Eclipse TE2000-U stereo microscope with a digital camera attached (Nikon).

GspS activity assay.

To 200 mM HEPES (pH 7.3) containing 1.5 mM NADH, 2 mM phosphoenolpyruvate (PEP) and 2 mM $MgCl_2$ was added a substrate solution of 40 mM GSH, 8 mM ATP and 0-6.4 mM spermidine (or biotin-spm), 11.4 U ml^{-1} pyruvate kinase, 48.96 U mL^{-1} lactate dehydrogenase. GspS (2.5 mg ml^{-1}) was then added to initiate the enzyme activity assay. The consumption of NADH was measured by fluorescence excitation at 340 nm and emission at 460 nm to determine the degree of product formation.

An Additional References.

[12] Blagbrough, I.S. & Geall, A.J. *Tetrahedron* **2000**, 56, 2449-2460.
